# *Leptospira* Contamination in Household and Environmental Water in Rural Communities in Southern Chile

**DOI:** 10.3390/ijerph110706666

**Published:** 2014-06-26

**Authors:** Claudia Muñoz-Zanzi, Meghan R. Mason, Carolina Encina, Angel Astroza, Alex Romero

**Affiliations:** 1 Division of Epidemiology and Community Health, School of Public Health, University of Minnesota, 1300 S. Second St., Suite 300, Minneapolis, MN 55454, USA; E-Mail: maso0299@umn.edu; 2 Institute of Animal Pathology, Austral University of Chile, Valdivia, Chile; E-Mails: carolina.encina.o@gmail.com (C.E.); astroza.a@gmail.com (A.A.); alexromero@uach.cl (A.R.)

**Keywords:** *Leptospira*, water, environment, PCR, rural

## Abstract

Leptospirosis is a zoonosis of global distribution that affects tropical and temperate areas. Under suitable conditions, *Leptospira* can survive in water and soil and contribute to human and animal infections. The objective of this study was to describe the presence of pathogenic *Leptospira* in peri-domestic water samples from rural households in southern Chile. Water samples, including puddles, containers, animal troughs, rivers, canals, and drinking water were collected from 236 households and tested for *Leptospira* using a PCR assay targeting the *lipL32* gene. Evidence of *Leptospira* presence was detected in all sample types; overall, 13.5% (77/570) samples tested positive. A total of 10/22 (45.5%) open containers, 12/83 (14.5%) animal drinking sources, 9/47 (19.1%) human drinking sources, and 36/306 (19.3%) puddles tested positive. Lower income (OR = 4.35, *p =* 0.003), increased temperature (OR = 1.23, *p <* 0.001), and presence of dogs (OR = 15.9, *p =* 0.022) were positively associated with positive puddles. Increased number of rodent signs was associated with positive puddles in the household (OR = 3.22); however, only in the lower income households. There was no association between PCR positive rodents and puddles at the household level. Results revealed the ubiquity of *Leptospira* in the household environment and highlight the need to develop formal approaches for systematic monitoring.

## 1. Introduction

Leptospirosis, caused by exposure to pathogenic species of the spirochete bacteria *Leptospira*, has been classified as an emerging zoonotic disease of global distribution and concern [1,2]. People can become infected after exposure to water or soil contaminated with *Leptospira* shed in urine of infected animals [3]. The complex *Leptospira* transmission cycle includes rodents as well as domestic and wild animal as potential hosts that shed the bacteria in the urine for variable periods of time. Dogs, for example, shed *Leptospira* for at least 4 weeks after an experimental infection [4]. Rodents, in particular rats, act as efficient reservoirs that can shed *Leptospira* for considerably long periods of time [5]. The ability of the bacteria to persist for months in sufficiently warm and moist environments provides continued opportunities for human infection [6,7]. Leptospirosis can be asymptomatic in humans, cause flu-like symptoms, or produce more serious clinical conditions such as jaundice, renal failure, aseptic meningitis, pulmonary hemorrhaging and death [8]. Globally, annual burden of leptospirosis is estimated to be about 1.03 million cases with 58,900 deaths [9], but poor diagnostic tools and reporting mechanisms in areas of the world where the disease is most prevalent, likely contribute to an underestimation of the true burden of disease [1].

In tropical climates, the persistence of *Leptospira* in the environment is facilitated by flooding after periods of heavy rainfall where the warm, humid and suitable water and soil is abundant [5]. These deluges of rainfall often produce high incidence rates of human leptospirosis caused by contact with contaminated flood waters [10,11,12]. The association between contact with environmental water and the risk of *Leptospira* infection is less clear in temperate climates. Environmental exposure sources implicated in human leptospirosis cases in temperate regions have included tap water, stagnant pools and wells, waste water, and recreational waters [13,14]. In rural populations in particular, human leptospirosis has historically been attributed to direct contact with infected livestock urine through animal caretaking [15]. However, the importance of environmentally acquired leptospirosis has been noted as well [16,17].

Assessment of the presence of *Leptospira* in the environment has been greatly facilitated by the advancement of molecular methods [18]. The continued refinement and expansion of PCR methods have allowed for more specific and rapid amplification of pathogenic *Leptospira* in a variety of environmental samples, making it possible to identify contaminated soil and water sites [19,20]. Several studies have made use of these molecular tools for the detection of pathogenic *Leptospira* in environmental water samples with the purpose of documenting sources of potential human exposure risk. Reports have documented presence of *Leptospira*, detected by PCR, in soil and water samples from various sources and in different geographical areas including puddles and uncovered drainage systems in urban areas of Philippines, Japan [21], and Malaysia [22], as well as in puddles, irrigation areas, river and other open water sources in rural areas of Malaysia [23], Philippines [21], and Hawaii [24]. In Peru, a study was conducted that compared the presence of *Leptospira* in open gutters, puddles, streams, and underground water sources across rural and urban communities. In certain public market sites, 67.9% of puddles and gutters contained *Leptospira* DNA [19]. In the context of a larger project aimed at investigating the eco-epidemiology of endemic leptospirosis in temperate climates, the objective of this study was to describe presence of pathogenic *Leptospira* in water sources from the peri-domestic environment in eight farm and village communities from Southern Chile.

## 2. Experimental Section

### 2.1. Study Site and Population

This study was conducted in the Los Rios Region of Chile which is located in the south-central area of the country. The climate is characterized as temperate rainforest with annual cumulative rainfall of 2588 mm but can range from 1200 mm in the central valley to 5500 mm in the Andes Mountains. Average temperature in summer is 17 °C and in winter is 8 °C [25]. The moderate temperatures and high rainfall contribute to the presence of *Leptospira* in the study area, as evidenced by widespread animal infection [26,27,28], and low but endemic levels of human cases. Although under-diagnosed, and unknown for the study region, the annual incidence rate of reported leptospirosis cases in Chile is between 0.14 and 0.20 per 100,000 persons [29]. Evidence of prior exposure to *Leptospira* is notably more common, with sero-prevalence estimates recorded as high as 22% in certain high-risk populations in the region [30].

Between November 2010 and April 2012, a total of 280 households from eight communities in the Los Rios region were identified and enrolled in the study. Data collection was performed from spring through early fall each year, yielding two summer sampling seasons (January–April) and two spring sampling seasons (August–December). Households were selected based on representativeness of the community in which they resided and their willingness to participate. Study population included eight communities in total, four farm communities (*n =* 146 households in total) and four rural villages (*n =* 134 households in total). Farms were singular dwellings on plots of land, where household residents collected their own water for drinking and household activities. Rural villages were considered to be located away from major population centers but with closely spaced households where often a single, communal water source was accessed by community members. The study protocol was approved by the Ethics Committee of Austral University in Chile and by the University of Minnesota Institutional Review Board (No. 0903M62042).

### 2.2. Household Survey and Meteorological Data

A questionnaire was administered to the head of the each household to collect data on demographic, environmental, and behavioral characteristics of the household. Information obtained relevant to this study included household socio-demographic characteristics, drinking water sources, waste disposal practices, and the presence of animals in the household and peri-domestic area. A detailed description of the household characteristics has been previously reported [28].

Measures of daily rainfall (mm) for the duration of the study and a 70-day buffer on each side were obtained from the National Aeronautics and Space Administration (NASA) Tropical Rainfall Measuring Mission (TRMM) dataset (Washington, DC, USA) compiled with 3B42 algorithm, version 7. Each community was ascribed precipitation data based on its position within the 0.25 by 0.25 degree spatial grid used by NASA databases, resulting in 6 NASA dataset locations for the eight communities. These data were downloaded using the MIRADOR search tool and were provided in 3-h increments from which a daily measure of total precipitation was calculated, as well as for the 7 days and 30 days prior to water sampling. A similar approach was used to obtain surface temperature (K) from the Global Land Data Assimilation System (GLDAS) Noah model dataset version L4 (NASA, Washington, DC, USA) compiled for the same duration of time. These measurements were also provided in 3-h increments which were then averaged to calculate surface temperature estimates for the day of, as well as the 7 days and 30 days prior to water sampling.

### 2.3. Water Sample Collection

Household environmental water samples were primarily obtained from outside of the housing structure, within the peri-domestic environment. Water samples were collected from pails, buckets, large bins, trash cans, small streams, ditches, puddles and other standing water sources that could be accessed by domestic animals, livestock, and rodents. At least 50 mL of water, up to 1 L when possible, was collected from each water source using sterile technique. Samples were placed in Whirl-Pak bags (Nasco, Fort Atkinson, WI, USA), transported, and stored at 4 °C until processing for DNA extraction. The date, place of collection, and volume obtained for each sample were also recorded.

Samples were classified into one of five sample types: puddles, containers, animal troughs, rivers or irrigation channels, and drinking water sources. A puddle sample type was considered to be standing water that was most likely the result of rainfall but also included more permanent sources of standing water such as ponds or large ditches. Containers were debris found around the household areas and included, but were not limited to, buckets, pails, jars, barrels, and old tires. Drinking water samples could have come from wells, springs, or taps as reported by the head of household.

### 2.4. Rodent Trapping and Sample Collection

In order to evaluate the potential association between rodents and the presence of *Leptospira* in household environmental water, rodents were captured at each household and their kidneys tested for *Leptospira* carriage using PCR methods. The specific trapping procedures were described previously [28]. Briefly, rodents were captured over the course of 3 nights during the same time period when water samples were collected by placing traps within each house and in the peri-domestic area. The study protocol was approved by the Bioethics Committee for the Use of Animals in Research from Austral University in Chile (No. 01/09) and the IACUC from the University of Minnesota (No. 0904A63201). In addition to rodent trapping, a proxy measure for rodent abundance, defined as number of rodent signs, was obtained for each household as previously reported [28]. The number of rodent signs was the sum of the affirmative responses by the head of the household to the following evidence of rodent presence in the dwelling: rodent sightings, feces, urine, gnawed wood, chewed boxes, marks on the walls, rodent noises, or gnawed food.

### 2.5. Detection of Leptospira by PCR

Twenty ml of each water sample was centrifuged at 8000 × g for 10 min. The pellets were lysed immediately to begin the extraction process using a commercial kit (QIAamp DNA Stool Mini Kit, Qiagen, Hilden, Germany) according to the manufacturer’s instructions. DNA elution was performed with 50 µL of buffer AE. DNA extraction from 30 mg of kidney tissue was carried out using a commercial kit (E.Z.N.A^®^ Tissue DNA Kit, Omega Bio-Tek, Norcross, GA, USA) according to the manufacturer’s instructions. DNA elution was performed with 200 µL of elution buffer allowing at least 5 min of incubation. All samples were tested using a PCR targeting the *lipL32* gene using the previously published primers LipL32-45F (5'-AAG CAT TAC CGC TTG TGG TG-3') and LipL32-286R (5'-GAA CTC CCA TTT CAG CGA TT-3') which is conserved among pathogenic *Leptospira* species [31]. The PCR reactions were performed in 25 µL mixture containing 3 μL of template, 0.25 μM of each primer, 0.625 U of GoTaq Flexi DNA polymerase in 1× Green Buffer GoTaq (Promega, Madison, WI, USA), 2.5 mM MgCl_2_, 0.8 mM dNTPs (Promega), and 400 ng/mL BSA (BioLabs, Ipswich, UK). All samples were tested in duplicate. Cycle conditions included an initial denaturation step at 95 °C for 5 min, followed by 40 cycles at 94 °C for 1 min, 57 °C for 1 min and 72 °C for 1 min, and a final elongation step of at 72 °C for 10 min. Each amplification run contained a negative control, consisting of water and a positive plasmidial control. The PCR products obtained were separated on 1.5% (*w/v*) agarose gel, stained with Gel Red (GelRed™, Biotium Inc., Hayward, CA, USA) and purified using a commercial kit (E.Z.N.A^®^ Gel Extraction Kit, Omega Bio-Tek). Prior evaluation of detection limit revealed that a minimum concentration of 10^3^ cells was consistently detected by the protocol. For quality control purposes, sequences were obtained (Macrogen Inc., Seoul, Korea) and used in a BLAST search of GenBank to confirm similarity to *Leptospira* spp. sequences.

### 2.6. Statistical Analysis

Distributions of *Leptospira* positive samples were described by water sample type and community type using frequencies and chi-square tests or Fisher’s exact tests as appropriate. Formal multivariable statistical analysis to examine factors associated with *Leptospira* presence was carried out for puddle samples type only because they are in locations shared by people and potentially *Leptospira*-infected animals and because of the importance of puddle contamination for direct exposure risk associated with daily outdoor activities by household members. Analysis was done at the household level based on presence or absence of positive puddle samples using regression analysis for a binomial outcome with a random effect for community using the lme4 R package [32]. In order to account for the varying number of samples obtained at each household and that more than one sample from a household could test positive, the outcome corresponded to a two-column matrix where the first column indicated the number of positive puddle samples and the second column was the number of negative samples for each household. Univariable logistic regression was initially done for all variables of interest. Multivariable logistic regression was used to identify variables that jointly predicted whether a household had a positive puddle sample. A manual backward stepwise procedure was used for model selection based on significance of the variable (α = 0.05), whether it improved model fit as assessed by log-likelihood and Akaike Information Criteria, while assessing the need to include potential confounders. Interaction terms were examined among all independent variables, with special emphasis on the potential modifying effect of rainfall, temperature, and community type. A separate regression analysis was performed on the households with puddles samples in which rodents were also captured to examine the specific association between presence of *Leptospira* positive rodents and presence of positive puddle samples, while also adjusting for other household and meteorological factors. For the final models, odds ratios (OR) and 95% confidence intervals (CI) were obtained by exponentiation of the appropriate regression coefficients using a standard approach. R version 2.15 was used for all descriptive and regression analyses (R Development Core Team, Vienna, Austria).

## 3. Results

Of the 280 participating households, 236 (84.3%) contributed at least one water sample for analysis, resulting in a total of 570 water samples. On average, farms had the greatest number of water samples collected per household (2.62). Slightly fewer samples were obtained in the rural villages (2.17 per household). Overall, 13.5% (77/570) of the environmental water samples were identified as PCR positive (Table 1). BLAST search of these sequences confirmed non-saprophytic *Leptospira* species.

**Table 1:** Results of PCR testing for pathogenic *Leptospira* in household and environmental water samples in four rural villages and four farm communities from Los Rios region, Chile (2010–2012).

Water Sample Type	Proportion of Samples Positive for *Leptospira*			
	Rural Villages	Farms	Total	*p*-Value ^a^
**Puddles**	16.7% (14/84)	21.6% (22/102)	19.3% (36/186)	0.51
**Canals/rivers**	2.9% (3/41)	1.6% (1/62)	3.9% (4/103)	0.30
**Containers**	12.0% (6/50)	11% (10/91)	11.3% (16/141)	1.0
**Animal troughs and drinking sources**	14.3% (4/28)	14.6% (8/55)	14.5% (12/83)	1.0
**Human drinking water**	0% (0/10)	19.1% (9/47)	15.8% (9/57)	0.60
**Total**	12.7% (27/213)	14.0% (50/357)	13.5% (77/570)	0.75

^a^ *p*-value from the statistical comparison of the proportions of positive samples between rural villages and farms.

### 3.1. Samples from Open Containers and Animal Drinking Sources

Samples collected from open containers and debris in the peri-domestic area were among the most commonly collected sample type after puddles, with 102 of the 280 households (36.4%) contributing at least one container sample. A total of 141 container samples were collected (Table 1). Notably, most of the positive container samples (10 out of the 16 positive samples, Table 1) came from a single farm community. In this community, 10/22 (45.5%) samples tested positive and two households had 2/2 positive samples and 2/3 positive samples, respectively. Of the 102 households, 13 (12.8%) had positive container samples with no suggestion of any particular household or environmental factors significantly associated with increased number of positive samples (data not shown).

Samples from animal drinking sources or troughs were obtained in 60 of the 236 households (25.4%) that contributed water samples, mostly in farms. From a total of 83 samples, 12 (14.5%) were positive for pathogenic *Leptospira* with no significant difference in the proportion positive between farms (14.3%) and villages (14.6%) (*p =* 1.0). More households had positive troughs samples in Year 2 compared with Year 1 of sampling (OR: 5.47, 95% C.I.: 1.48–20.29, *p =* 0.011). In addition, increased average temperature 30 days prior to sample collection was positively associated finding households with positive trough sample (OR for a one unit increase in temperature: 1.48, 95% C.I.: 1.07–2.06, *p =* 0.020).

### 3.2. Human Drinking Water Sources, Rivers, and Irrigation Channels

On farms, 9/47 (19.1%) of human drinking water sources tested positive for *Leptospira*, compared to 0% in rural villages (*p =* 0.60, Table 1). The nine drinking water samples that tested positive for *Leptospira* in the farms were collected from wells. Eight of the households that had positive well water samples also cited wells as their primary drinking water source. Only one of the well water-drinking households reported boiling water “most of the time” before consuming it, four households occasionally boiled their water, and the other three households never boiled water. Only two households of the eight that had wells that tested positive reported chlorinating their water, and did so only occasionally. Overall, four of the 103 (3.9%) water samples from rivers and irrigation channels tested positive for *Leptospira* with no difference between community types (*p =* 0.30).

### 3.3. Puddles from the Peri-Domestic Environment

Puddles were the most commonly collected sampled, with 46.8% (131/280) of all enrolled households providing at least one sample and 55.6% (131/236) of the households that provided at least one water sample. Number of puddle samples per household collected ranged from 1 to 4; however, in the large majority of the households (123) one or two samples were collected. A total of 36/306 (19.3%) of the puddle samples tested positive (Table 1) and at least one puddle sample tested positive in 26% (34/131) of the households. There was a wide range in the proportion of positive puddles in each of the eight communities (3% to 44%). In the univariable analysis (Table 2), lower income was the only household variable associated with increased presence of *Leptospira*-contaminated puddles (OR = 3.03, *p =* 0.017). Of the environmental variables, temperature, in particular 7-days prior to collection was associated with increased detection of households with positive samples (OR = 1.17, *p =* 0.007). Precipitation one-month prior to collection was negatively associated with positive households (OR = 0.99, *p =* 0.005). Multivariable model selection yielded a parsimonious model, adjusted for precipitation, where the beneficial effect of higher household income (OR = 0.23, 95% C.I.: 0.09–0.60, *p =* 0.003) and the positive association with average temperature 7 days prior to collection (OR = 1.23, 95% C.I.: 1.09–1.39, *p <* 0.001) were maintained. An additional significant variable in the model was the presence of dogs suggesting that the odds of contaminated puddles were greater in households with dogs compared with household without dogs (OR = 15.9, 95% C.I.: 1.48–171.2, *p =* 0.022).

**Table 2:** Univariable mixed-effects logistic regression analyses for the association between household level factors and the presence of pathogenic *Leptospira* in puddle water samples collected from the peri-domestic environment, Los Rios region, Chile.

Variables	Proportion Positive ^a^ Households	OR (95% CI)	*p*-value
**Community Type**			
Rural Villages	25.0%	–	
Farms	26.7%	1.10 (0.31, 3.93)	0.887
**Monthly Income (USD)** ^c^			
<$208	47.6%	–	
≥$208	21.8%	0.33 (0.13, 0.82)	0.017
**Sampling Year**			
1st	17.0%	–	
2nd	31.0%	1.89 (0.53, 6.72)	0.322
**Sampling Season**			
Summer/Fall	31.4%	–	
Spring	22.5%	0.48 (0.17, 1.42)	0.185
**Sanitary Disposal**			
Septic Tank/System	27.5%	–	
Latrine/None	22.5%	1.16 (0.47, 2.86)	0.746
**Own ≥ 1 Dog**			
No	11.1%	–	
Yes	27.0%	5.49 (0.59, 51.49)	0.136
**No. rodent signs**			
0–1	32.7%	–	
≥2	21.1%	0.58 (0.26, 1.26)	0.166
**No. Livestock**			
0 Animals	14.3%	–	
1–30 Animals	33.3%	2.51 (0.65, 9.68)	0.182
>30 Animals	21.3%	1.65 (0.38, 7.12)	0.504
	**Mean in negative, positive households** ^b^	**OR (95% CI)** ^d^	
**No. Household Members**	4.34, 3.85	0.86 (0.67, 1.10)	0.235
**No. Domestic Species**	3.68, 3.85	1.20 (0.92, 1.55)	0.183
**No. Rodents Captured**	1.33, 0.65	0.73 (0.51, 1.04)	0.084
**Rainfall (mm)**			
30 Days prior to Sampling	71.37, 45.72	0.99 (0.98, 1.00)	0.005
7 Days prior to Sampling	16.01, 13.03	1.01 (0.99 1.03)	0.600
Day of Sampling	1.81, 1.10	0.97 (0.89, 1.06)	0.458
**Average Temperature (°C)**			
30 Days prior to Sampling	12.13, 13.40	1.15 (1.02, 1.30)	0.026
7 Days prior to Sampling	12.58, 14.30	1.17 (1.05, 1.31)	0.007
Day of Sampling	12.66, 14.93	1.13 (1.03, 1.25)	0.012

^a^ Positive is defined here as a household with at least one PCR positive puddle water sample; ^b^ Mean value of the variable among negative (first value) and positive (second value) households; ^c^ $100,000 Chilean pesos = $208 U.S. dollars; ^d^ OR was calculated for a one unit increase.

A total of 69 households had rodents captured as well as puddle samples collected. Analysis of this subset of households revealed that among 24 households with at least one *Leptospira* positive rodent, only one household (4.2%) had positive puddle samples. Conversely, of the 45 households where all rodents tested negative, 12 (26.7%) had at least one puddle positive for *Leptospira* (*p =* 0.087). Furthermore, regression modeling examining the association between number of rodents signs, as a proxy for rodent presence, and positive puddle samples suggested household income as an effect modifier (*p =* 0.006 for the interaction term). Among higher income households, fewer positive puddles were found in households that reported >2 rodent signs (OR: 0.31, 95% C.I.: 0.12–0.79). Conversely, among lower income households, reporting >2 rodent signs suggested a positive association with presence of contaminated puddles in the household (OR = 3.22, 95% C.I.: 0.96–42.41).

## 4. Discussion and Conclusions

This study further demonstrates the ubiquity of *Leptospira* in water from the household and the peri-domestic environment in general, as suggested by PCR detection of a DNA target present in pathogenic *Leptospira* species [33]. Results showed evidence of contamination, at various levels, in all sample types including puddles, containers and debris, animal drinking troughs, rivers and canals, and household drinking water (Table 1). An increasing number of studies are investigating presence of *Leptospira* in environmental samples and have reported efforts to detect pathogenic *Leptospira* DNA in water (mostly rivers, lakes, other water sources in public places as well as drinking water sources) from non-tropical [34] and tropical settings [35,36]. Notably, use of molecular approaches revealed community and sample type differences in presence and concentration of *Leptospira*, where puddle and gutter samples from urban areas had much higher levels of contamination than well and stream water samples from a rural village [19]. Differences in sampling designs and PCR protocols do not allow comparisons between studies or with the 13.5% of PCR positive samples found in the present study; however, it highlights the current need and the interest in understanding, more formally, the role of the environment in leptospirosis transmission and as a public health risk.

Contact with contaminated water or soil has been documented as one of the most frequent modes of exposure among the sporadic cases identified in non-tropical countries. In Italy, 53% of cases reported an occupation that involved contact with animals and/or polluted waters and among 78% of cases with established mode of exposure, water exposure was the likely mechanism [13]. In Germany, 59% of cases were reported as environment or water-related; either through occupational or recreational activities [37]. Outbreaks occur less frequently than in tropical countries and when do, they are also often associated with water exposure [17,38,39,40]. The public health significance of the current findings (13.5% of total samples with a PCR positive result, 19.3% of puddles from the peri-domestic environment, and 26% of households with at least one positive sample) for the local population is not known. Accurate ascertainment of local incident cases is not available; however, based on sero-surveys in the same study population showing average sero-prevalence of 6% (range from 2.6% to 10.3%) [41], environmental contamination is likely to cause some level of human infection that is not recognized or identified due to mild illness and/or lack of proper diagnostic resources.

As opposed to saprophytic leptospires, pathogenic species have a life cycle that includes both environmental and animal infection phases [5]. Qualitative PCR results of the study samples did not allow further determination of potential sources of the environmental contamination; however, epidemiological analysis of the data suggested a positive association between presence of positive puddles in the household and increased number of rodent signs as well as with the presence of dogs. Number of rodent signs was previously found to be a good predictor of rodent captures [28]. Rodents are well recognized reservoirs of infection and are often assumed responsible for contamination of soil and water through shedding of *Leptospira* in the urine [5,42]. However, this positive association in the study data was only found in the lowest income households. Furthermore, examination of the data revealed that many households in the higher income category had PCR positive samples but no PCR positive rodents or reports of increased number of rodent signs. Although this was not manifested in the statistical analysis that included livestock-related variables, because higher income households were more often found in farms, we can speculate that presence of other animals or environmental contamination mechanisms play a role in explaining the observed data. Among the variables representing presence of domestic animal species, presence of dogs was associated with increased likelihood of positive puddles in the households. Dog ownership is common in the study population with 84% of the households reporting owing 1 to 10 dogs. Estimate of sero-prevalence in dogs was 26% [43] suggesting that some dogs with active shedding could be contaminating the peri-domestic environment [44]. The detection of positive samples in well samples is noteworthy but not unexpected as wells were commonly poorly built and unprotected which exposed them to the elements, including potential runoff from contaminated surface water and/or direct animal exposure. Pathogenic *Leptospira* is able to adapt for survival in the environment under suitable conditions [45]; however, our results, based on DNA detection, cannot differentiate viable from non-viable bacteria. Ongoing work includes sequencing of the *secY* gene in a subset of positive samples, which has been shown to be more suitable for species classification and phylogenetic analysis [46]. Future work should involve intensive parallel water and carrier testing and detection of isolates in order to investigate specific sources of contamination.

Temperature is a known key factor in the survival of leptospires in the environment [5]. Although optimal growth temperatures near 28–30 °C, bacteria has been detected in samples obtained from water as cool as 9.5 °C [47]. Shedding of pathogenic *Leptospira* under temperature conditions that are suitable for survival is conducive to indirect transmission between hosts and persistence of infection in the area. This study found evidence of a positive association between temperature prior to sample collection and evidence of *Leptospira* presence in both puddles (Table 2) and animal troughs. In tropical regions, warmer temperatures often come with additional rainfall in the hot and humid “rainy seasons” which may contribute to flooding, increased runoffs, and spread of *Leptospira* contamination of the environment [48]. It was hypothesized that rainfall prior to sample collection would be a contributing factor in disseminating urine from infected animals and [5,49], therefore, show as a positive association with positive samples; however, we did not find an association. Limiting factors in examining this association was the cross-sectional nature of the study and using rainfall predictors at specific time points (30 days, 7 days, and 0 day prior to collection). Furthermore, this descriptive report did not include formal investigation of other possible predictors such as leptospirosis in domestic animals and other community-level and landscape variables.

Longitudinal and systematic monitoring of environmental contamination is needed to improve our understanding of the role of the local environment in maintenance of infection in a community and to identify triggers of increased incidence in people and animals. A major problem with attempting to apply environmental risk assessment approaches has been the limitations of using traditional culture methods which are time and resource consuming, have low sensitivity, and are often hindered by the dominance of saprophytic bacteria [35]. However, the field may see a significant expansion as modified culture methods are being proposed [21,22] and molecular methods are able to provide quicker qualitative and quantitative results [50].
